# Keratoconus with Central Serous Chorioretinopathy: A Rare Combination

**DOI:** 10.1155/2020/8816449

**Published:** 2020-07-14

**Authors:** Anastasia Tsiogka, Apostolos Gkartzonikas, Konstantinos Markopoulos, Iordanis Georgiou, George L. Spaeth

**Affiliations:** ^1^Department of Ophthalmology, 401 General Military Hospital, 3 P. Kanellopoulou Street, Athens 11525, Greece; ^2^Department of Ophthalmology, Wills Eye Hospital, 840 Walnut St, Philadelphia, PA 19107, USA

## Abstract

Keratoconus and central serous chorioretinopathy are two rare diseases. They can occur together in some individuals. We report a case of a 48-year-old man, who presented to our clinic with decreased visual acuity on his left eye. Physical examination, biomicroscopy, corneal topography, and optical coherence tomography revealed keratoconus and central serous chorioretinopathy. We discuss the possible connection between these two conditions.

## 1. Introduction

Keratoconus causes visual loss due to increasing irregular corneal astigmatism and affects 86 in 100,000 people [1]. It is an idiopathic, noninflammatory, progressive corneal ectasia characterized by bilateral, usually asymmetric thinning of the cornea [2]. The onset typically is in teenage years, and it is much less common in middle-aged and elderly individuals [3, 4]. While it often presents as an isolated condition, keratoconus may also be associated with many systemic disorders and/or ocular diseases [5].

Central serous chorioretinopathy (CSC) is a major cause of vision threat among middle-aged male individuals [6]. It is a secondary idiopathic serous neurosensory detachment, believed to be due to a leakage of fluid from the choroid through tight junctions between adjacent retinal pigment epithelial cells (RPE) [7]. Its precise pathogenesis is unknown, but there are some possible risk factors relating to the occurrence of CSC, such as H. pylori infection, steroid use, sleep disorders, autoimmune disease, psychotropic medication use, and type-A behavior [8]. To the best of our knowledge, no association between CSC and corneal diseases has been described. Here, we present the case of a patient with keratoconus and central serious chorioretinopathy (CSC).

## 2. Case Report

### 2.1. History

A 48-year-old male was referred to our ophthalmology clinic with decreased visual acuity on the left eye. His medical history included Crohn's disease since 2005, seasonal allergic rhinoconjunctivitis, and prostate cancer status post prostatectomy in 2016. He had been treated with methylprednisolone intermittently therapy for the last 4 years. Five years ago, he had an episode of episcleritis treated with dexamethasone (1%) drops. He was very anxious. His family history was unremarkable.

### 2.2. Measurements

#### 2.2.1. Entrance Tests, Refraction, and Binocularity

Best corrected distance visual acuity was 10/10 in the right eye and 2/10 in the left eye (Snellen chart). Ocular motility was normal. Visual fields were intact to confrontation visual field exam (Donders' test). His pupils were equal, round, and reactive to light, with no afferent pupillary defect. Color vision discrimination, using the Ishihara color vision test, showed normal color vision in each eye. The red-cap color comparison was equal between the two eyes. Automated keratometry revealed of -1.00 sph and -1.50 cyl at 90° in the right eye and -5.00 cyl at 120° in the left eye.

#### 2.2.2. Biomicroscopy

The eyelids, eyelashes, conjunctiva, iris, and anterior chamber appeared normal. Anterior segment biomicroscopy showed mild corneal thinning inferior to the pupil in the left eye. There were no other signs of keratoconus (Fleischer's ring, Vogt's striae, Rizzuti's sign, oil drop). There was no stromal edema or corneal opacities in either eye. He had incipient posterior subcapsular cataract in both eyes. Fundus examination of both eyes was normal. Vitreous, optic nerves, vasculature, and peripheral retina in both eyes were unremarkable and normal for his age. The intraocular pressure (as measured by Goldmann applanation tonometry) was 30 mmHg in the right eye and 28 mmHg in the left eye.

#### 2.2.3. Corneal Topography and Optical Coherence Tomography (OCT)

**Figure 1:**
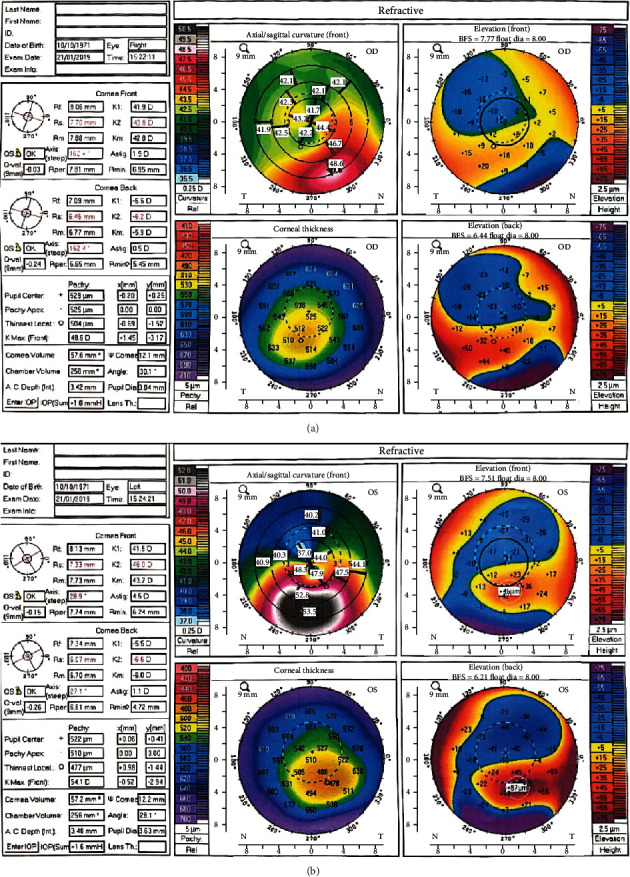
Corneal topography of the right eye with subclinical keratoconus (a) and corneal topography of the left eye showing keratoconus (b).

The anterior surface corneal topography of the left eye showed normal K1 and K2 values, but the difference between Kmax-K2 was >1D. For the right eye, the simulated minimum keratometry reading had normal K1 and K2 values and the difference between Kmax-K2 was abnormal as the left eye. Secondary, Kmax (do)-Kmax (so) was >2D. Corneal pachymetry measurements at the thinnest locations were 477 microns and 504 microns for the left and right eye, respectively (Figure 1).

**Figure 2:**
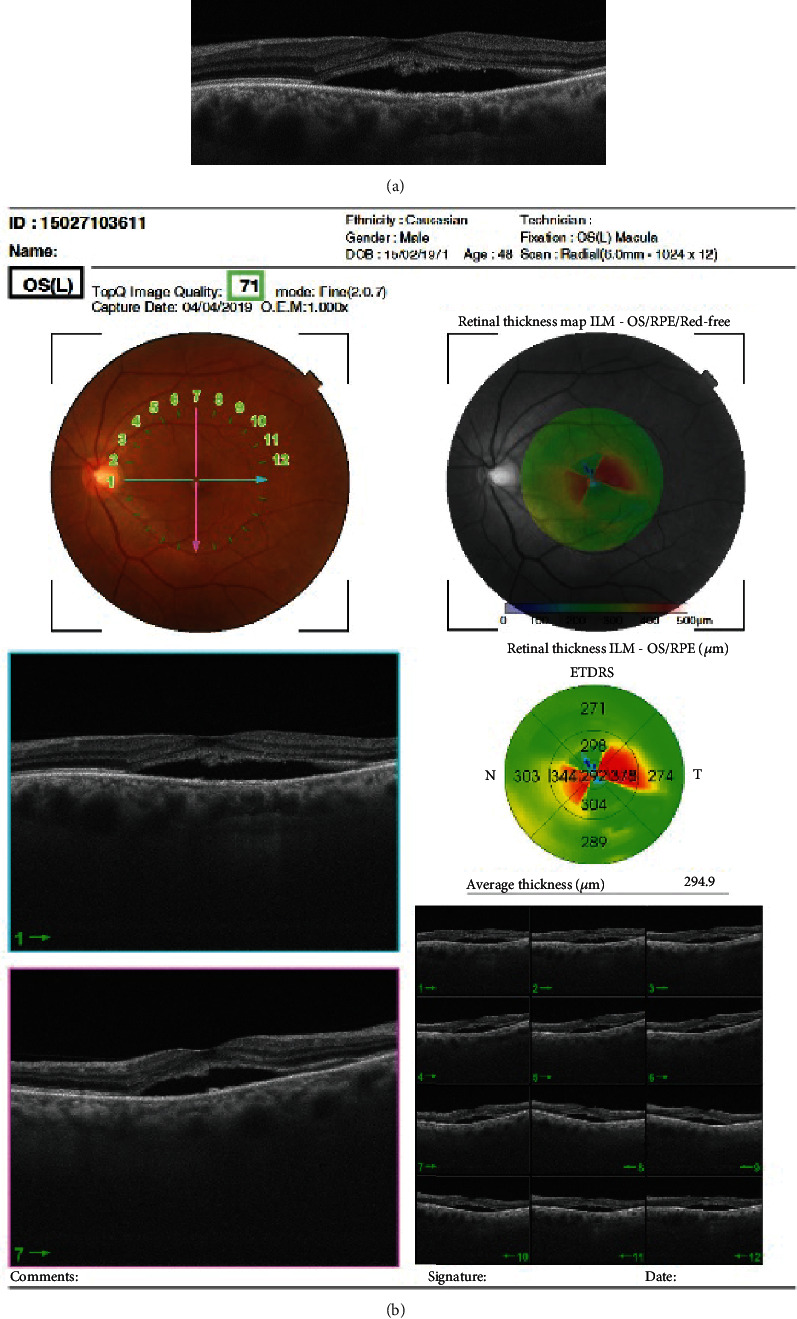
OCT horizontal high-resolution image (a) and retinal thickness map (b) of the left eye showing central serous chorioretinopathy.

The OCT examination demonstrated fluid accumulation between the interdigitation zone (IZ) and the retinal pigment epithelium (RPE) as well as increased choroidal thickness. The junction between photoreceptor inner and outer segments (IS/OS) was not detected in the detached neurosensory retina. The outer photoreceptor layer of the detached neurosensory retina above the clear subretinal space was irregularly thickened and granulated (Figure 2(a)). The retinal thickness ILM was increased in the horizontal meridian (Figure 2(b)).

Clinical findings were characteristic of keratoconus, central serious chorioretinopathy, and elevated intraocular pressure, as a result of steroid therapy.

### 2.3. Treatment

Aldosterone antagonists are effective in decreasing subretinal fluid and improving visual acuity in patients with CSC ^9^. We prescribed the selective aldosterone antagonist eplerenone which is associated with a favorable side effect profile. Beta blockers are not the most potent medication of IOP lowering, but they are efficacious, well tolerated by most patients and a low cost-effective choice, making them a good choice as a first-line agent in case of the absence of any contraindications. They induce b receptor blockage which lowers aqueous humor formation and secretion especially during daytime [9, 10]. Timolol is one of the most popular and prescribed antiglaucoma beta blocker agents, as a first-line drug in most forms of open angle glaucoma and ocular hypertension [11]. Our patient had elevated IOP due to chronic use of systemic steroids as part of treatment for his Crohn's disease. Given the necessity for ongoing steroid therapy, we elected to prescribe timolol to lower IOP (probably steroid responder). Spectacles were, also, prescribed for keratoconus to improve his vision [3].

## 3. Discussion

This case report demonstrates the coexistence of keratoconus and central serous chorioretinopathy to the left eye of a 48-year-old Caucasian male with Crohn's disease. Keratoconus and CSC are diseases that affect different parts of the eye.

Keratoconus may be associated with many systemic disorders such as diabetes, asthma, Down syndrome, collagen vascular disease, and sleep apnea [12]. There are some strong associations between keratoconus and several autoimmune diseases and allergic disorders [13]. Furthermore, patients with inflammatory bowel diseases may have an increased risk of keratoconus [14].

The main treatment of inflammatory bowel disease is corticosteroids. They are administered topically, orally, or intravenously and rapidly and consistently improve moderate to severe active ulcerative colitis and Crohn's disease [15]. There is no known association between corticosteroids and keratoconus. In vitro trials of dexamethasone on human cornea showed that dexamethasone significantly increases keratocyte proliferation, but it also induces apoptosis of cultured keratocytes at any concentration [16]. Keratocyte apoptosis is associated with keratoconus, in which keratocyte density is lower compared to healthy controls [17, 18]. On the other hand, central serous chorioretinopathy has been associated with most routes of steroid administration. The majority of the literature on CSC and steroid medication suggests a significant association [19]. At the current time, the pathophysiology of CSC is poorly understood [11].

To our knowledge, there is no direct association between these two conditions. The subfoveal choroid is diffusely thickened in patients with CSC likely because of the choroidal vascular dilatation [20, 21]. In addition, the subfoveal choroid in keratoconus eyes is thicker than that in healthy population [22, 23].

A genetic disorder could better explain the coexistence of keratoconus and central serous chorioretinopathy, especially in predisposed individuals. Keratoconus is associated with extensive alteration in the expression of genes coding collagen type IV, fibronectin, laminin, lysyl oxidase, and tissue inhibitor of metalloproteinase 3 (TIMP-3) involved in cell-matrix, cell-cell interactions, massive changes of the cytoskeleton, extracellular matrix remodeling, and transmembrane signaling [24, 25].

The TIMPs are multifunctional proteins, with different cellular effects which are not well understood. TIMP-3 is the only TIMP protein that binds tightly to the extracellular matrix [26]. Immunohistochemistry showed TIMP3 immunostaining was dense, specifically localized in Bruch's membrane and some choroidal vascular basement membranes [27]. Increased expression of TIMP3 could contribute to the activation of apoptotic cell death processes through restructuring of the architecture, and disruption of photoreceptor—matrix interactions. Altered expression of TIMP-3 could modify the RPE basement membrane, causing variation in the pigment epithelial integrity. These observations could explain the disturbance of the outer blood–retinal barrier [28].

Reduced expression of some proteins to RPE due to genetic disorders could explain a relationship between the two diseases, potentially reflecting a dysfunction of epithelial layers and basement membranes [29–31]. More studies are necessary to elucidate a possible connection between these diseases.

## 4. Conclusion

According to the literature, there is no known association between keratoconus and CSC. Genetic predisposition seems to be a plausible mechanism to explain the occurrence of both conditions on the same individual. More studies are necessary to elucidate the possible connection between these diseases.

## Abbreviations

CSC: Central serous chorioretinopathy
IZ: Interdigitation zone
IOP: Intraocular pressure
IS/OS: Inner and outer
OCT: Optical coherence tomography
RPE: Retinal pigment epithelium.

## References

[1] Ferdi A. C., Nguyen V., Gore D. M., Allan B. D., Rozema J. J., Watson S. L. (2019). Keratoconus Natural Progression: A Systematic Review and Meta-analysis of 11 529 Eyes. *Ophthalmology*.

[2] Spaeth G. L., Danesh-Meyer H., Goldberg I., Kampik A. (2011). *Ophthalmic Surgery: Principles and Practice E-Book: Expert Consult-Online and Print*.

[3] Rabinowitz Y. S. (1998). Keratoconus. *Survey of Ophthalmology*.

[4] Gordon-Shaag A., Millodot M., Shneor E. (2012). The epidemiology and etiology of keratoconus. *International Journal of Keratoconus and Ectatic Corneal Diseases*.

[5] Hauser J. W. M. A. (2012). The genetics of keratoconus: a review. *Reproductive System & Sexual Disorders*.

[6] Daruich A., Matet A., Dirani A. (2015). Central serous chorioretinopathy: recent findings and new physiopathology hypothesis. *Progress in Retinal and Eye Research*.

[7] Guyer D. R., Yannuzzi L. A., Slakter J. S., Sorenson J. A., Ho A., Orlock D. (1994). Digital indocyanine green videoangiography of central serous chorioretinopathy. *Archives of Ophthalmology*.

[8] Liu B., Deng T., Zhang J. (2016). Risk factors for central serous chorioretinopathy: a systematic review and meta-analysis. *Retina*.

[9] Dada T., Ichhpujani P., Spaeth G. (2011). Pearls in Glaucoma Therapy.

[10] El-Khamery A. A.-E., Mohamed A. I., Swify H. E. H., Mohamed A. I. (2017). Cost-effectiveness of glaucoma management with monotherapy medications in Egypt. *Journal of advanced pharmaceutical technology & research*.

[11] Tătaru C. P., Purcărea V. L. (2012). Antiglaucoma pharmacotherapy. *Journal of Medicine and Life*.

[12] Woodward M. A., Blachley T. S., Stein J. D. (2016). The association between sociodemographic factors, common systemic diseases, and keratoconus: an analysis of a nationwide heath care claims database. *Ophthalmology*.

[13] Nemet A. Y., Vinker S., Bahar I., Kaiserman I. (2010). The association of keratoconus with immune disorders. *Cornea*.

[14] Tréchot F., Angioi K., Latarche C. (2015). Keratoconus in inflammatory bowel disease patients: a cross-sectional study. *Journal of Crohn's and Colitis*.

[15] Katz J. A. (2004). Treatment of inflammatory bowel disease with corticosteroids. *Gastroenterology Clinics of North America*.

[16] Bourcier T., Borderie V., Forgez P., Lombet A., Rostène W., Laroche L. (1999). In vitro effects of dexamethasone on human corneal keratocytes. *Investigative Ophthalmology & Visual Science*.

[17] Kim W.-J., Rabinowitz Y. S., Meisler D. M., Wilson S. E. (1999). Keratocyte apoptosis associated with keratoconus. *Experimental Eye Research*.

[18] Soiberman U., Foster J. W., Jun A. S., Chakravarti S. (2017). Pathophysiology of Keratoconus: What Do We Know Today. *The open ophthalmology journal*.

[19] Nicholson B. P., Atchison E., Idris A. A., Bakri S. J. (2018). Central serous chorioretinopathy and glucocorticoids: an update on evidence for association. *survey of ophthalmology*.

[20] Akkaya S. (2018). Macular and peripapillary choroidal thickness in patients with keratoconus. *Ophthalmic Surgery, Lasers and Imaging Retina*.

[21] Gutierrez-Bonet R., Ruiz-Medrano J., Peña-Garcia P. (2018). Macular choroidal thickening in keratoconus patients: swept-source optical coherence tomography study. *Translational Vision Science & Technology*.

[22] Maruko I., Iida T., Sugano Y., Ojima A., Sekiryu T. (2011). Subfoveal choroidal thickness in fellow eyes of patients with central serous chorioretinopathy. *Retina*.

[23] Kuroda S., Ikuno Y., Yasuno Y. (2013). Choroidal thickness in central serous chorioretinopathy. *Retina*.

[24] Nielsen K., Birkenkamp-Demtro¨der K., Ehlers N., Orntoft T. F. (2003). Identification of differentially expressed genes in keratoconus epithelium analyzed on microarrays. *Investigative Ophthalmology & Visual Science*.

[25] Khaled M. L. (2019). In search of genetic mutations for familial keratoconus.

[26] Brew K., Dinakarpandian D., Nagase H. (2000). Tissue inhibitors of metalloproteinases: evolution, structure and function. *Biochimica et Biophysica Acta (BBA) - Protein Structure and Molecular Enzymology*.

[27] Vranka J. (1996). TIMP3 expression in the human retina and choroid. *Investigative Ophthalmology and Visual Science*.

[28] Jones S. E., Jomary C., Neal M. J. (1994). Expression of TIMP3 mRNA is elevated in retinas affected by simplex retinitis pigmentosa. *FEBS Letters*.

[29] Turksen K., Aubin J. E., Sodek J., Kalnins V. I. (2017). Localization of laminin, type IV collagen, fibronectin, and heparan sulfate proteoglycan in chick retinal pigment epithelium basement membrane during embryonic development. *Journal of Histochemistry & Cytochemistry*.

[30] Eandi C. M., del Priore L. V., Bertelli E., Ober M. D., Yannuzzi L. A. (2008). Central serous chorioretinopathy in patients with keratoconus. *Retina*.

[31] Sawaguchi S., Yue B. Y., Sugar J., Gilboy J. E. (1989). Lysosomal enzyme abnormalities in keratoconus. *Archives of Ophthalmology*.

